# Pneumonia in Children

**Published:** 1890-04

**Authors:** 


### PNEUMONIA IN CHILDREN.

Septic pneumonia may occur at labor, but the more common forms are croupous pneumonia and the broncho pneumonia, which not infrequently follows whooping-cough. In twelve cases of whooping-cough in children, all were complicated by pneumonia. The whooping-cough was treated with steam inhalations of liquor sodii boratis compositus (Dobell's solution) and antipyrine. With this, plenty of fresh air was given, and the whooping-cough disappeared. In these twelve cases there was pneumonia, of different grades of severity. Treatment in these cases was local and constitutional. Internally, they were given aromatic spirits of ammonia and whiskey freely. They were then put in a mustard bath 90 degrees until the skin became distinctly red (ten to fifteen minutes), and at the same time cold cloths were applied to the head. They were then taken out and a flannel bandage dipped in tap water, was wrapped around the chest and back, and then hot blankets and bottles to the limbs, and plenty of brandy to stimulate. Following such a bath the temperature fell, and sleep supervened. Antipyrine was given in small doses, as a sedative, but opium was not used. Most of these cases made good recoveries. For the cases that became chronic, flannel jackets, double ply, with a layer of oil silk between, were used next the skin in preference to poultices, as this caused them to be less liable to shock from sudden changes of temperature. Chloride of ammonia, with licorice, was given to liquefy the expectoration. Every one of the twelve cases recovered. In children, pneumonia is lobular and not lobar, as a general rule, and there is little or no dullness to be detected by percussion, owing to the small size of the area infected.—*Davis; Times and Register.*

